# Annotations

**Published:** 1902-09-06

**Authors:** 


### Annotations.

### The Sanitary Advantages of Shaving.

The beard and moustache, if bacteriologists are to be believed, form the happy hunting ground of numerous microbes. Perfect cleansing of hair is always attended with considerable difficulty. There is therefore much sound scientific reason for the custom of shaving. We have often been surprised and amused at the elaborate care with which certain Esau-like surgeons seek to render aseptic their finger nails while a few minutes later their unwashed and unprotected hirsute appendages are waving over an open abdomen, and even in some instances coming almost if not actually into contact with the patient. We hear that the command has gone forth in Germany that all operating surgeons must shave. Whether this be so or not we cannot say, but certainly it is based on right principles. The inconsistencies of scientists are stumbling blocks for the true disciple, and the indiscretions of surgeons often bring surgery into disrepute. Certainly if the principles of aseptic surgery are to be carried out in practical work mere æsthetics must, if needs be, be set aside and surgeons must be prepared to signify their thoroughness by sacrificing facial adornment, and demonstrating the sanitary advantages of shaving.

### The Army Medical Service.

A careful article in the *Times*, born somewhat out of date, perhaps, but interesting from the fulness of its comments, gives a general estimate of the reforms which are likely to be produced by the new Royal Warrant for pay and promotion in the medical service of the Army. For purposes of comparison, it is assumed that the average gross income of 50 per cent. of medical men starting general practice is (*a*) at starting, £500; (*b*) after three years, £600; (*c*) after ten years, £800 to £900; (*d*) after twenty years, £900 to £1,200 per annum; the professional expenses knocking these sums down to net incomes of £333, £400, £600, and £800 respectively. We do not pretend to guess whence the writer gets his figures, but starting on this basis he finds it an easy task to demonstrate that the balance is in favour of the army officer, especially when the pension of £1 a day after twenty years' service is taken into consideration. There is one drawback, as everyone who has considered the matter knows full well, and that is India, the allowances to medical officers serving in that part of the world being, as he says, "wholly inadequate." That the immediate effect of the warrant has been, and for a time will continue to be, to induce students to compete for commissions, and thus to raise the whole tone of the service we cannot doubt. But the permanence of this improvement will depend upon the spirit in which the provisions contained in the warrant are fulfilled, and in regard to that matter those whose faith in Royal Warrants has been shaken by harsh experience recall to our attention the facts that Lord Herbert of Lea's Royal Commission in 1858 recommended the granting of study leave—one of the inducements now again put forward—and that although officially adopted as a principle it has practically never been granted to the present day. Lord Camperdown's Committee, again, in 1889, recommended a grant of three months' study leave every seven years, a rectification of the Indian allowances, and a shortening of the term of foreign service. Yet these things are not. The first fruits of the promises given have been good, but their permanence will depend upon the Government keeping faith.

### Another Danger to South Africa.

Mr. Jonathan Hutchinson again returns to the charge and warns all those whom it may concern that a very pressing danger now awaits South Africa in regard to the extension of leprosy. During the war the inland fish trade has been almost suspended, the railways being needed for other purposes; but now that labourers are flocking to the mines and farmers to their homesteads, the demand for food will cause badly cured salt fish conveyed in sacks to be sent inland in large quantities, and with it the infection of leprosy. In past years, he says, it has been the mining population and the agricultural labourers who have supplied Robben Island and Emjanyana with their inmates, and they will do so again at increased rates unless something be done to control the trade of the Cape Colony fisheries and prevent any fish leaving their establishments except such as is in a state of sound preservation. Precautions to ensure that the so-called "salt-fish" should be properly cured with good salt and plenty of it would probably not appreciably increase the price of fish inland, while it would, on the other hand, he says, save the large amount of money annually expended in maintaining the miseries of Robben Island. How one wishes that all this were demonstrable! That, as Mr. Hutchinson says, nine out of ten of the victims of leprosy will assure one that prior to their own illness they had never seen a case of the disease; that children brought up in leper homes do not contract the disease; that nurses, ward servants, and doctors alike escape; that it is exceedingly rare for husband and wife to suffer together, although they may have cohabited for ten or twenty years; and that all inoculation experiments have failed may be admitted, and undoubtedly such facts go to suggest that those who think to put a stop to leprosy by segregation of the sufferers are on the wrong track. On the other hand, not one, nor even the whole assembly, of these facts goes to prove that leprosy is caused by eating fish.

### Sanatoria fcr the Consumptive Poor.

Dr. Frank Birchell, at the recent Annual Congress of the Royal Institute of Public Health at Exeter, advocated the provision and maintenance of sanatoria by sanitary and Poor-law authorities. Certainly there are immense numbers of patients who in consequence of poverty, or surroundings, or the advanced stage of the disease, are now unable to receive adequate treatment or adopt appropriate precautions, and hence become a serious menace to their friends and a source of danger to the public. The extinction of consumption cannot be accomplished as long as we neglect to care for the disease-disseminating destitute poor.

